# Association of Dietary Intake of Vitamin A With Adolescent Hypertension: A Cross‐Sectional Study Based on NHANES 1999–2018

**DOI:** 10.1002/fsn3.4643

**Published:** 2024-12-30

**Authors:** Yukang Mao, Lin Chen, Yuli Wang, Lida Wu, Guidong Xu, Xiangqing Kong, Chao Chen, Jiayi Weng

**Affiliations:** ^1^ Department of Cardiology The Affiliated Suzhou Hospital of Nanjing Medical University, Suzhou Municipal Hospital, Gusu School, Nanjing Medical University Suzhou China; ^2^ Department of Cardiology The First Affiliated Hospital of Nanjing Medical University Nanjing China; ^3^ Department of Cardiology Nanjing First Hospital Affiliated to Nanjing Medical University Nanjing China

**Keywords:** adolescent, cross‐sectional study, hypertension, NHANES, vitamin a

## Abstract

We aimed to investigate the association between dietary intake of vitamin A and risk of hypertension during adolescence. We interrogated the National Health and Nutrition Examination Survey (NHANES) database, from which individual‐level data on dietary intake of vitamin A were garnered from 13,909 adolescents (aged 10–19 years) participating in the 1999–2018 study cycle. After dividing vitamin A intake into four quartiles, we leveraged weighted multivariate logistic regression to investigate the association of vitamin A intake with hypertension by each quartile, with the restricted cubic spline (RCS) curve plotted to assess the nonlinearity of association. Additionally, we performed subgroup analysis to examine whether gender remarkably affects vitamin A's effect on hypertension. Of all the adolescent participants, 1477 (10.6%) were found to have hypertension. Following thorough adjustments for confounding factors, per 1‐SD increment in vitamin A intake was associated with a 23%, 26%, and 31% reduction in the risk of hypertension for the 2nd, 3rd, and 4th quartiles, respectively. Consistently, the RCS curve indicated that the risk of adolescent hypertension presented a decreasing trend as vitamin A intake creeped up. Intriguingly, gender‐stratified subgroup analysis demonstrated that the observed association between vitamin A and adolescent hypertension was more pronounced in boys. Together, our findings outlined vitamin A as a protective dietary factor against hypertension among US adolescents. When using vitamin A supplements for preventing hypertension, boys may gain more practical benefits.

## Graphical abstract

We confirmed for the first time that there exists a significantly inverse association of vitamin A intake with adolescent hypertension independently of multiple confounding factors in the US general population, with the association found to be more pronounced in boys. In other words, boys may benefit more from using vitamin A supplements for preventing hypertension during their adolescence.

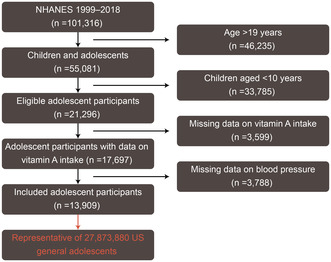

## 1 Introduction

Hypertension, also termed chronically elevated systemic blood pressure (BP), is posing an overwhelming threat to human health, contributing substantially to cardiovascular disability and mortality. Globally, ~8.5 million people died of hypertension or its complications in 2015, with approximately 88% of them coming from low‐ and middle‐income countries (Zhou et al. 2021). Notwithstanding the wide recognition of hypertension as a chronic disease mostly occurring in middle‐aged or elderly populations, a sharp increase in pediatric individuals (both children and adolescents) diagnosed to have hypertension has been witnessed by the past two decades (Song et al. 2019). This is quite concerning as poorly controlled BP during childhood and adolescence oftentimes persists into adulthood and becomes the major source of a higher cardiovascular burden during the lifetime. Unlike the consensus of adulthood hypertension definition, where a BP value of 140/90 mmHg is set as a uniform threshold, childhood and adolescent hypertension is diagnosed only when BP level exceeds 95th percentiles by age, sex, and height (Sun et al. 2017). Under normal circumstances, the incidence of hypertension in the pediatric population varies with advancing age, which begins to climb since the early stage of puberty and presents a constantly increasing trend throughout the entire adolescence before reaching its peak level at the end of puberty (Song et al. 2019). It is postulated that such phenomenon is physiologically attributable to gonadal maturation and sex hormone secretion and, as such, adolescents appear to be more vulnerable to hypertension than children do (Ewald and Haldeman Ph 2016; Shankar et al. 2005). In addition, overweight/obesity related to undesirable lifestyles (e.g., overeating or sedentary behaviors) is deemed another important predisposing factor for adolescent hypertension (Sorof and Daniels 2002).

Vitamin A, technically referring to retinol and its derived compounds, is a kind of fat‐soluble vitamin that serves as a crucial modulator of cell proliferation, differentiation, and apoptosis. Biologically, vitamin A is central to the normal body growth and development of children and adolescents, while also facilitating the maintenance of vision, reproductive function, and immune response (Blaner 2019; von Lintig 2012). Because of incapacity to synthesize vitamin A in the human body, the only pathway through which vitamin A can be obtained is diet, specifically two types of vitamin A‐rich foods, including animal‐derived foods such as animal liver, eggs, fish liver oil, and plant‐based foods like carrots and pumpkins (Villamor and Fawzi 2005). While overall, there is highly consistent and compelling evidence that vitamin A exerts BP‐reducing actions—predominantly through repressing oxidative stress, improving endothelial function, or modulating the activity of renin–angiotensin system (RAS)—in various animal models of hypertension (Abbasian, Alavi, and Roohbakhsh 2023; Młynarska et al. 2024), whether people who are at risk of developing hypertension can gain adequate benefits from vitamin A supplements remains debatable. Some studies strongly support that an increased intake of vitamin A is associated with a lower risk of hypertension (Li, Chen, and Zhang 2019; McCarron et al. 1984; Zhang et al. 2021), whereas some others did not (Albuquerque, Diniz Ada, and Arruda 2009; Llopis‐González et al. 2015). Of note, the abovementioned studies primarily focus on adulthood hypertension, and little is known about the case in pediatric individuals. A case–control study involving 164 Chinese children aged 6–12 years reported an inverse association between the mRNA level of retinol acyltransferase, a molecular marker of vitamin A storage function, in peripheral blood leukocytes and BP levels, despite failure to observe a significant difference in serum vitamin A between hypertensive and nonhypertensive subjects (Liang et al. 2018). This perhaps hints at potential clinical relevance of vitamin A in the management of childhood and adolescent hypertension; however, population‐based data are sparse thus far. To fill this knowledge gap, we planned to take advantage of the large sample representative of the US general population deposited within the National Health and Nutrition Examination Survey (NHANES) database.

## 2 Methods

### 2.1 Study Population

**FIGURE 1:**
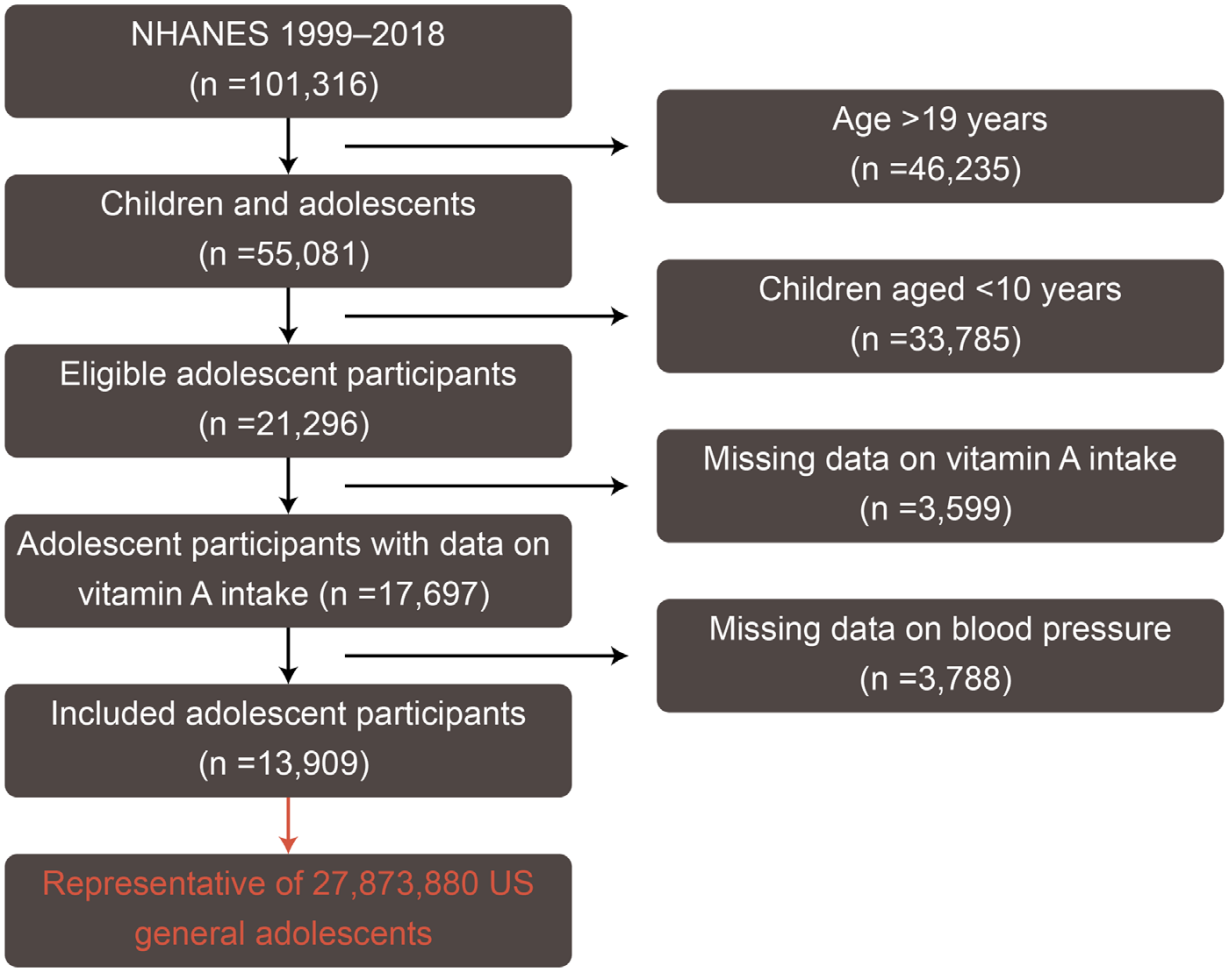
A detailed workflow of study population inclusion.

The NHANES is an ongoing research project initiated by the National Center for Health Statistics (NCHS), with an intention of assembling nationwide information on nutritional and health status of contemporary US civilians (https://www.cdc.gov/nchs/nhanes/index.htm). In our study, cross‐sectional data of 101,316 participants coming from ten consecutive study cycles of the NHANES (1999–2018) were downloaded from the NHANES website. For data processing, we began by ruling out data of nonpediatric individuals (age > 19 years; *n* = 46,235). Next, given the vast majority of hypertensive cases in the pediatric population occur during adolescence, we only retained the data pertaining to adolescents (age ≥ 10 and ≤ 19 years, *n* = 21,296), thus excluding those pertaining to children (age < 10 years; *n* = 33,785). After further excluding participants without available data on dietary intake of vitamin A (*n* = 3599) and hypertension status (*n* = 3788), the remaining 13,909 were ultimately included in the analyses, which is capable of representing ~30 million US adolescents because of full consideration of sampling weights in NHANES study design. The NHANES study protocols have gained approval from the National Center for Health Statistics ethics review board, with written informed consent garnered from all participants. A step‐by‐step workflow of study participant enrollment is presented in Figure 1.

### 2.2 Assessment of Dietary Intake of Vitamin A

Dietary intake data on vitamin A were collected during a face‐to‐face interview, specifically the What We Eat in America survey launched by the US Department of Agriculture (USDA) and US Department of Health and Human Services. In that survey, all NHANES participants were requested to undergo two interviews where they recalled and reported all foods and beverages they consumed in the past 24 h before the interview (from midnight to midnight). The first interview was hosted by trained interviewers at a mobile examination center, with a subsequent interview conducted via a phone call 3–10 days later. To assess the nutrients, energy, and other food ingredients that a diet contains, dietary intake data were coded and processed according to the USDA's Food and Nutrient Database for Dietary Studies. The intake values pertaining to vitamin A were respectively calculated from both dietary recalls and were averaged to represent the overall vitamin A intake (Huang et al. 2020; Shen et al. 2020). For more details on the acquisition and quantification of vitamin A intake, please see the protocols available at the NHANES website (https://wwwn.cdc.gov/Nchs/Nhanes/2003‐2004/DR1IFF_C.htm).

### 2.3 Assessment of Adolescent Hypertension

Because of the natural impact of rapid body development during adolescence on BP levels, the major diagnostic criterion for adolescent hypertension is quite different from that used for adulthood hypertension in clinical practice. It is inappropriate to diagnose adolescent hypertension based on whether systolic/diastolic blood pressure (SBP/DBP) exceeds a specific value, usually 140/90 mmHg. Instead, an adolescent would only be deemed hypertensive when his/her BP level exceeds those of 95% of gender‐ and height‐matched peers, which aligns with most guidelines (Lurbe et al. 2009; Xi et al. 2016). At examination, experienced medical workers used a mercury sphygmomanometers to measure BP levels (Wu et al. 2023). After a 5‐min seated rest, three BP readings were obtained at 30‐s intervals and were averaged to generate a final BP value (Wu et al. 2022). More detailed information on BP measurement is available on the NHANES website (http://www.cdc.gov/nchs/data/nhanes/pe.pdf).

### 2.4 Covariates

Given the multifaceted etiologies of adolescent hypertension, we accounted for multiple variables that may serve as potential confounders in the analyses. Race/ethnicity was self‐reported according to five categories provided by NHANES investigators (non‐Hispanic White, non‐Hispanic Black, other Hispanic, Mexican American, and others). Family income (< $2000 or ≥ $2000) was used to quantify economic income status. A higher family income usually indicates a better household economic condition (Wu et al. 2023). NHANES utilized several standardized food composition database—one of which is the USDA Food Composition Database—to provide nutritional information for each food item, including protein, fat, carbohydrates, and others. The energy contained in each food item is typically expressed with kilocalories (kcal) and is added together to generate a total energy intake value (Mao et al. 2024). Height and weight were measured during physical examination, with BMI calculated accordingly by dividing weight (kg) by the square of height (m^2^). A BMI of 25–29.9 kg/m^2^ and 30 kg/m^2^ or greater was considered overweight and obese, respectively (Li et al. 2022). The estimated glomerular filtration rate (eGFR) was estimated using a formula launched by the Chronic Kidney Disease Epidemiology Collaboration, where age, sex, race/ethnicity, and serum creatinine (SCr) were adjusted (Li et al. 2021). Fasting blood glucose (FBG), glycated hemoglobin (HbA1c), triglyceride (TG), total cholesterol (TC), low‐density lipoprotein cholesterol (LDL‐C), high‐density lipoprotein cholesterol (HDL‐C), hemoglobin (Hb), and red blood cell (RBC) counts in the peripheral blood were measured after an 8‐h fasting (Mao et al. 2024). Individuals who provided a self‐reported diagnosis were considered to have diagnosed diabetes mellitus (DM), while those lacking a previous diagnosis but conforming to at least one of the following criteria according to laboratory tests can be categorized as having undiagnosed DM: (1) an HbA1c level of 6.5 or greater, (2) an FPG level of 7.0 mmol/L or greater, and (3) a plasma glucose level of 11.1 mmol/L or greater during a 2‐h oral glucose tolerance test (OGTT) (Cheng et al. 2019).

### 2.5 Statistical Analysis

Because of the use of complex multistage sampling design in NHANES, we accounted for the sampling weights for generating accurate effect estimates for data spanning different study cycles (Chen et al. 2020, 2018; Johnson et al. 2013). Comparisons of continuous characteristics between participants grouped by hypertension status and quartiles of vitamin A intake were conducted with Student's *t*‐test and one‐way ANOVA, respectively, and chi‐square test was used for comparing categorical characteristics. Given vitamin A intake is a continuous variable, we divided it into four quartiles when performing the analyses. This was not based on previous literature but rather determined by the vitamin A intake of all populations included in this study. To infer the independent association of vitamin A intake and hypertension, we established a series of logistic regression models; that is, an unadjusted model and two models with multiple covariate adjustments (Model I and II). In Model I, age, sex, and race/ethnicity were adjusted, and Model II included all covariates in Model I plus family income, total energy intake, BMI, eGFR, and DM. We also modeled vitamin A intake using restricted cubic splines (knots at the 10th, 50th, and 90th percentiles) to assess the shape of association between vitamin A intake and risk of hypertension. To further examine whether gender has a significant impact on this association, stratified analyses were performed. R software 4.1.6 (http://www.R‐project.org, The R Foundation, Vienna, Austria) was used for implementing all data analyses, with a two‐tailed *p*‐value < 0.05 deemed statistically significant.

## 3 Results

### 3.1 Baseline Characteristics of Study Participants

The overall characteristics of 13,909 adolescents aged 10–19 years are described in Table 1. Of all the participants, the prevalence of hypertension was 10.6%. We noted that participants with hypertension tended to be younger boys or girls, consistent with previous observations that the incidence of pediatric hypertension usually varies with age. A higher proportion of non‐Hispanic Black and individuals with poorer household economic status appeared in the hypertension group. By contrast, no significant difference in gender and total energy intake was observed between two groups. As for clinical characteristics, compared to their nonhypertensive counterparts, hypertensive adolescents were inclined to be overweight/obese, have a more adverse lipid profile (higher TG, TC, LDL‐C, and lower HDL‐C), and present poorer blood glucose tolerance (higher FBG, HbA1c, and prevalence of DM). Also, slightly higher levels of eGFR and RBC count appeared in the hypertension group, whereas Hb level remained comparable between both groups. In addition, significant differences can be observed among participants grouped by quartiles of vitamin A intake, in which those in higher quartiles were more likely to be younger, males, non‐Hispanic Whites, and lean and, more importantly, have lower BP levels (Table S1).

**TABLE 1:** Baseline characteristics of study population grouped by hypertension status.

Variable	Overall (*n* = 13,909)	Nonhypertension (*n* = 12,432)	Hypertension (*n* = 1477)	*p*
Age, %				< 0.001***
10–14 years	61.37 [58.42, 64.33]	60.78 [59.63, 61.93]	66.80 [63.84, 69.77]	
15–19 years	38.63 [36.58, 40.67]	39.22 [38.07, 40.37]	33.20 [30.23, 36.16]	
Gender, %				0.08
Female	49.53 [47.07, 51.99]	49.89 [48.67, 51.11]	46.25 [42.41, 50.09]	
Male	50.47 [47.92, 53.01]	50.11 [48.89, 51.33]	53.75 [49.91, 57.59]	
Ethnicity, %				0.02*
Non‐Hispanic White	58.38 [53.74, 63.02]	58.70 [56.18, 61.22]	55.50 [50.80, 60.20]	
Non‐Hispanic Black	14.30 [13.00, 15.61]	13.88 [12.38, 15.38]	18.21 [15.26, 21.16]	
Mexican American	12.99 [11.48, 14.50]	12.96 [11.35, 14.57]	13.26 [10.62, 15.90]	
Other Hispanic	6.49 [5.49, 7.48]	6.59 [5.56, 7.62]	5.55 [3.77, 7.34]	
Others	7.83 [6.89, 8.78]	7.87 [6.93, 8.81]	7.48 [5.42, 9.54]	
Family income, %				0.04
< $2000	16.55 [15.46, 17.63]	16.75 [15.57, 17.93]	19.85 [16.80, 22.89]	
≥ $2000	80.48 [76.31, 84.64]	83.25 [82.07, 84.43]	80.15 [77.11, 83.20]	
Energy, kcal	2077.37 [2055.80, 2098.95]	2077.92 [2055.69, 2100.15]	2072.41 [2009.53, 2135.28]	0.87
SBP, mmHg	106.77 [106.46, 107.08]	105.34 [105.07, 105.62]	119.83 [118.97, 120.68]	< 0.001***
DBP, mmHg	59.27 [58.82, 59.72]	58.42 [58.01, 58.84]	67.04 [65.81, 68.27]	< 0.001***
BMI, %				< 0.001***
Normal weight	74.95 [71.19, 78.71]	76.46 [75.27, 77.66]	61.95 [58.26, 65.63]	
Over weight	10.17 [9.36, 10.98]	9.12 [8.34, 9.89]	19.96 [17.03, 22.90]	
Obesity	14.76 [13.71, 15.82]	14.42 [13.49, 15.35]	18.09 [15.30, 20.89]	
eGFR	136.38 [135.67, 137.09]	136.23 [135.51, 136.96]	137.91 [136.31, 139.51]	0.04*
FBG, mmol/L	5.26 [5.21, 5.31]	5.25 [5.19, 5.30]	5.35 [5.27, 5.44]	0.03*
HbA1c, %	5.20 [5.18, 5.22]	5.20 [5.18, 5.21]	5.25 [5.20, 5.31]	0.04*
DM, %	0.90 [0.63, 1.18]	0.80 [0.56, 1.04]	1.88 [0.77, 2.98]	0.003**
TG, mmol/L	0.94 [0.91, 0.97]	0.92 [0.90, 0.95]	1.13 [0.99, 1.28]	0.005**
TC, mmol/L	4.10 [4.08, 4.12]	4.09 [4.06, 4.11]	4.21 [4.15, 4.27]	< 0.001***
HDL‐C, mmol/L	1.34 [1.33, 1.34]	1.34 [1.33, 1.35]	1.28 [1.26, 1.31]	< 0.001***
LDL‐C, mmol/L	2.29 [2.27, 2.32]	2.28 [2.25, 2.30]	2.48 [2.39, 2.56]	< 0.001***
RBC, × 10^9^/L	4.77 [4.75, 4.78]	4.76 [4.74, 4.77]	4.86 [4.83, 4.89]	< 0.001***
Hemoglobin, g/L	13.94 [13.89, 13.99]	13.93 [13.88, 13.99]	13.97 [13.87, 14.07]	0.43

*Note:* Continuous variables are presented as mean [95% CI], and category variables are presented as proportion [95% CI]. Comparisons of continuous and categorical variables between hypertension and nonhypertension groups were conducted using unpaired two‐tailed Student's *t*‐test and chi‐square test, respectively.

Abbreviations: BMI, body mass index; DBP, diastolic blood pressure; DM, diabetes mellitus; eGFR, estimated glomerular filtration rate; FBG, fasting blood glucose; HbA1c, glycated hemoglobin; HDL‐C, high‐density lipoprotein cholesterol; LDL‐C, low‐density lipoprotein cholesterol; RBC, red blood cell; SBP, systolic blood pressure; TC, total cholesterol; TG, triglyceride.

* *p* < 0.05.

** *p* < 0.01.

*** *p* < 0.001.

### 3.2 Association of Dietary Intake of Vitamin A and Adolescent Hypertension

**FIGURE 2:**
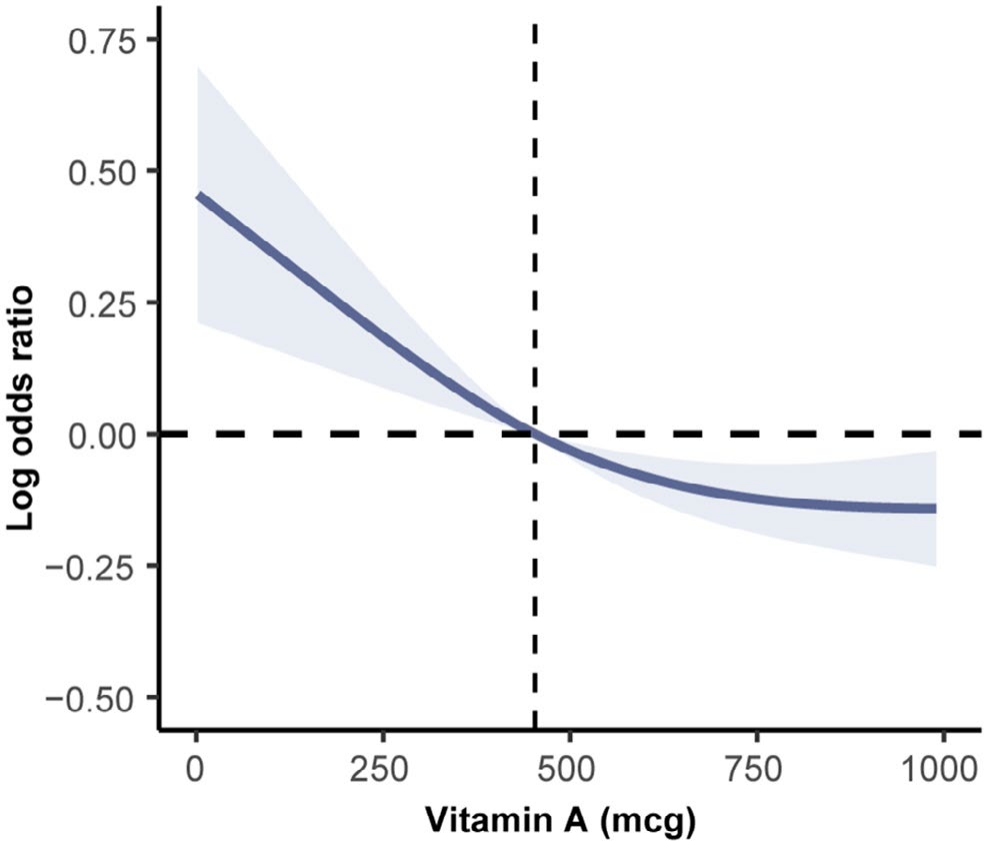
RCS regression analysis assessing the association between dietary intake of vitamin A and adolescent hypertension.

We performed a sampling‐weighted multivariate logistic regression analysis on the association of vitamin A intake with adolescent hypertension by different quartiles of vitamin A intake. Overall, we revealed that an increased intake of vitamin A was associated with a lower risk of adolescent hypertension, which remained consistent in both unadjusted and adjusted models (Table 2). In Model I, after controlling for age, sex, and race/ethnicity, we found that compared to the lowest quartile (Q1; considered as reference), the odds ratios (ORs) for adolescent hypertension in higher quartiles (Q2–4) were 0.77 (95% CI: 0.63–0.95), 0.75 (95% CI: 0.61–0.92), and 0.70 (95% CI: 0.57–0.87), respectively. After further adjustments of other confounders in Model II, including family income, total energy intake, BMI, eGFR, and DM, a more significant association was observed, where adolescents consuming more vitamin A daily had a 23% (OR: 0.77; 95% CI: 0.60–0.98; *p* = 0.001), 26%, and 31% lower chance of developing hypertension, respectively (Q2: 0.77 (95% CI: 0.60–0.98); Q3: 0.74 (95% CI: 0.56–0.98); Q4: 0.69 (95% CI: 0.52–0.92)). Having determined the inverse association between vitamin A intake and adolescent hypertension, we conducted RCS regression to further analyze the shape of association and found an inflection point located at a vitamin A intake of 466 mcg in the fitted curve. There was a linear dose–response association on the left side of the inflection point; however, when vitamin A intake exceeds a threshold value of 466 mcg, the decreasing rate of hypertension risk began to slow down, as evidence by the presence of a flatter curve on the other side of the inflection point (Figure 2).

**TABLE 2:** Weighted multivariate logistic regression analysis assessing the association of dietary intake of vitamin A with adolescent hypertension.

Vitamin A	Unadjusted model		Model I		Model II	
	OR [95% CI]	*p*	OR [95% CI]	*p*	OR [95% CI]	*p*
Q1	Reference	—	Reference	—	Reference	—
Q2	0.81 [0.66, 0.99]	0.04*	0.77 [0.63, 0.95]	0.02*	0.77 [0.60, 0.98]	0.04*
Q3	0.80 [0.65, 0.99]	0.04*	0.75 [0.61, 0.92]	0.01*	0.74 [0.56, 0.98]	0.04*
Q4	0.76 [0.61, 0.94]	0.01*	0.70 [0.57, 0.87]	0.001**	0.69 [0.52, 0.92]	0.01*

*Note:* Data are presented as OR [95% CI]. Model I was adjusted for age, sex, and race/ethnicity. Model II was adjusted for all covariates in Model I plus family income, total energy intake, BMI, eGFR, and DM.

Abbreviations: BMI, body mass index; CI, confidence interval; DM, diabetes mellitus; eGFR, estimated glomerular filtration rate; OR, odds ratio.

* *p* < 0.05.

** *p* < 0.01.

*** *p* < 0.001.

### 3.3 Gender‐Stratified Subgroup Analysis

**FIGURE 3:**
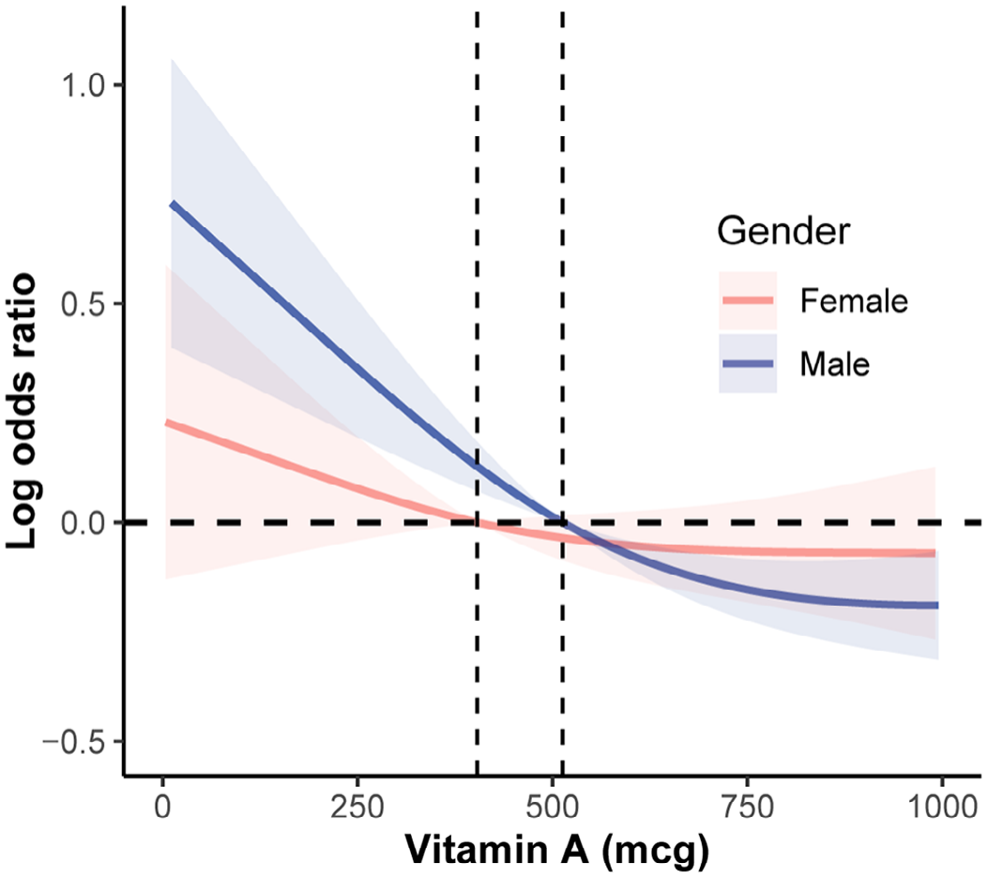
Gender‐stratified RCS regression analysis on the association between dietary intake of vitamin A and adolescent hypertension.

We next sought to decipher the potential influence of gender on the association between vitamin A intake and adolescent hypertension, as massive production of gonadal sex hormones is postulated to serve as an important aspect of hypertension etiology during adolescence according to the literatures. After gender stratification, the association between increasing quartiles of vitamin A intake and reduced risk of hypertension remained significant in boys—but not girls (Table 3), consistent with what was discovered in the gender‐stratified RCS curve, in which the magnitude of reduction in the risk of hypertension appeared to be more pronounced in boys rather than in girls as dietary intake of vitamin A gradually increased (Figure 3).

**TABLE 3:** Gender‐stratified logistic regression analysis assessing the association of dietary intake of vitamin A with adolescent hypertension.

Vitamin A	Unadjusted model		Model I		Model II	
	OR [95% CI]	*p*	OR [95% CI]	*p*	OR [95% CI]	*p*
Female						
Q1	Reference	—	Reference	—	Reference	—
Q2	0.86 [0.65, 1.15]	0.31	0.85 [0.64, 1.12]	0.25	0.82 [0.57, 1.17]	0.27
Q3	0.91 [0.68, 1.21]	0.50	0.87 [0.65, 1.17]	0.25	0.93 [0.63, 1.37]	0.7
Q4	0.93 [0.70, 1.24]	0.63	0.87 [0.65, 1.17]	0.35	0.81 [0.52, 1.27]	0.35
Male						
Q1	Reference	—	Reference	—	Reference	—
Q2	0.72 [0.53, 0.97]	0.03*	0.70 [0.51, 0.95]	0.02*	0.70 [0.48, 1.02]	0.06
Q3	0.67 [0.48, 0.93]	0.02*	0.64 [0.46, 0.89]	0.01*	0.57 [0.37, 0.87]	0.01*
Q4	0.59 [0.43, 0.81]	< 0.001***	0.58 [0.43, 0.78]	< 0.001***	0.57 [0.37, 0.88]	0.01*

*Note:* Data are presented as OR [95% CI]. Model I was adjusted for age, sex, and race/ethnicity. Model II was adjusted for all covariates in Model I plus family income, total energy intake, BMI, eGFR, and DM.

Abbreviations: BMI, body mass index; CI, confidence interval; DM, diabetes mellitus; eGFR, estimated glomerular filtration rate; OR, odds ratio.

* *p* < 0.05.

** *p* < 0.01.

*** *p* < 0.001.

## 4 Discussion

We performed a first‐of‐its‐kind cross‐sectional study looking into the effect of vitamin A on adolescent hypertension in the general population using the NHANES database. According to the results, we concluded that the risk of hypertension in adolescents decreased with increasing dietary intake of vitamin A and, more importantly, that boys may gain more benefits from vitamin A supplementation in terms of preventing hypertension. These results added new evidence to the management of hypertension from a perspective of diet and nutrition, while also heavily suggesting that gender be accounted for when formulating preventative strategies for adolescent hypertension based on vitamin A supplementation.

Hypertension has a high prevalence globally, constituting a highly prevalent chronic disease that can lead to the occurrence and progression of various cardiovascular and cerebrovascular diseases (Su, Kim, and Won 2021; Wang, Yang, and Fu 2021). According to a recent report, the prevalence of hypertension worldwide has reached over 30%, and in some developing countries, the prevalence of hypertension can even exceed 40% (Lu et al. 2024). Therefore, effective prevention and management of hypertension are of utmost importance for human health and life expectancy (Batubo, Moore, and Zulyniak 2024; Hu et al. 2024). In recent years, a growing body of evidence has supported the clinical relevance of focusing on adolescent hypertension with long‐term cardiovascular benefits (Anglum 2009; Pall, Kiss, and Katona 2012). Due to relative incompleteness of physical development in adolescents, the diagnostic criteria for adolescent hypertension in current clinical guidelines are markedly different from those for adulthood hypertension, that is, having a BP level exceeding the 95th percentiles by age, sex, and height (Sun et al. 2017). In this case, we adopted this approach to assess the outcome measure of adolescent hypertension. It is noteworthy that we determined which participants had adolescent hypertension based on BP data from all participants in the NHANES database, rather than identifying patients with adolescent hypertension among those included and excluded based on screening criteria in this study. Given the large number of participants included in NHANES, our diagnosis of adolescent hypertension is relatively objective and can reflect the prevalence of adolescent hypertension across the US. Actually, there have been studies conducted in the general population that explore the risk factors and treatment‐related targets for adolescent hypertension. Liang et al. (2022) research suggested that exposure to environmental pollutant acrylamide is intimately related to hypertension in adolescents. Importantly, the authors conducted a mediation analysis, with results indicating that TC mediates the relationship between acrylamide in the blood and the incidence of hypertension. Additionally, a Korean study indicated that postmenopausal women with a history of adolescent pregnancy have a markedly higher prevalence of hypertension compared to those without. After adequately controlling for age, lifestyle, socioeconomic factors, and some other known hypertension‐related risk factors, the authors found a significant association between adolescent pregnancy history and adolescent hypertension (Park et al. 2016). Mechanistically, a higher susceptibility caused by adolescent pregnancy is postulated to engage abrupt changes in hormones (especially estrogen), maternal age, socioeconomic factors (e.g., marital status), psychological distress, and other general hypertension‐related risk factors that may occur more easily than usual (e.g., morbid obesity, uncontrolled blood glucose). Of note, among the female participants included in this study, we found no case of adolescent pregnancy. Therefore, adolescent pregnancy was not outlined as a focus in our analysis.

Vitamin A, an essential nutrient, presents substantial benefits to human health when consumed in moderation (Abadie et al. 2023; Zhang et al. 2021). Different forms of vitamin A metabolites have been extensively reported to display consistently protective effects against hypertension in animal research. Postulated mechanisms behind these phenomena might engage several aspects. Central to vitamin A's beneficial role in improving hypertension may be its potent antioxidant capacity, as oxidative stress—biologically featured by an excessive production and/or insufficient scavenging of a kind of reactive oxygen species (ROS)—has long been known as a culprit of hypertension and vascular damage (Griendling et al. 2021). Through preventing ROS accumulation and sustaining a normal oxidation–reduction (redox) status, vitamin A enables recovery of the oxidative damage of blood vessel walls and preservation of the structural and functional integrity of the vascular endothelium, thereby reducing the risk of hypertension (Młynarska et al. 2024). In living beings, one of the most important biological processes depending on vitamin A is immune and inflammatory response, as evidenced by previous observations in rodents that both innate and adaptive immunities are disturbed in the setting of vitamin A deficiency (Mora, Iwata, and von Andrian 2008). We therefore speculated that immunomodulation may partially mediate the beneficial effects of vitamin A on BP, given chronic low‐grade systemic inflammation caused by an oversupply of pro‐inflammatory mediators is a hallmark of hypertension (Harrison et al. 2011); however, direct evidence, either preclinical or clinical, remains insufficient. Interestingly, vitamin A was also discovered to have a direct impact on the RAS, a well‐documented molecular cascade implicated in the initiation and development of hypertension (Arendse et al. 2019). In some early‐stage works, researchers detected a diminished expression of components pertaining to the conventional, pro‐hypertensive portion of the RAS, including renin, angiotensin‐converting enzyme (ACE), angiotensin (Ang) II, and its type 1 (AT1) receptor both in vivo and in vitro after treatment with all‐*trans* retinoic acid (atRA), an active metabolite of vitamin A (Dechow et al. 2001; Takeda et al. 2000). Subsequent to those findings, Zhong et al. further revealed that atRA administration can attenuate high BP of spontaneously hypertensive rats, without additional effects on normal BP of Wistar‐Kyoto controls. This was related to the upregulation of ACE2—a marker of the alternative, antihypertensive portion of the RAS—in the heart and kidney (Zhong et al. 2004). On the cellular level, vitamin A has been depicted as an important regulator of cell proliferation, differentiation, and migration, including vascular endothelial cells and smooth muscle cells, both of which are key cellular effectors involved in the fine‐tuning of vasomotor tone and, of course, BP homeostasis. Emerging research has added to new evidence that RA and its downstream signaling are vital for modulating vascular development and remodeling (Lai et al. 2003; Pawlikowski, Wragge, and Siegenthaler 2019; J. Zhang et al. 2019), offering some clues that vitamin A may ameliorate hypertension through acting on specific receptors expressed in those vascular cells. Yet, cell‐type‐specific gain‐ and loss‐of‐function experiments using conditional gene editing techniques would be necessary to clarify which cell types may be high‐priority targets of vitamin A. Similar to previous studies, in this study, we also obtained vitamin A intake from the NHANES public database (Liu et al. 2024; Song and Jiang 2023). We extracted the vitamin A content from two dietary interviews and ultimately calculated the average intake as the vitamin A intake for all participants included in the study, which was helpful for accurately estimating vitamin A intake. In fact, many studies based on the NHANES database have extensively explored the relationship between vitamin A treatment and various diseases and the occurrence of different diseases (Cheng et al. 2023; Hu et al. 2022). Our study provides effective clinical evidence for the potential therapeutic effects of vitamin A on adolescent hypertension, especially in boys, hoping to assist in the prevention and control of adolescent hypertension in clinical practice. Of note, the RCS curves revealed a nonlinear relationship with a downward trend that flattens around the zero log odds ratio, particularly highlighting a potential threshold effect at 466 mcg of vitamin A. This phenomenon may suggest a biological plausibility where higher doses of vitamin A could lead to saturation, some adverse effects, or even toxicity, hence blunting the beneficial impact of vitamin A supplement. Additionally, specific population characteristics, such as dietary habits, baseline vitamin A levels, or genetic variations in metabolism, may also be related to the presence of threshold.

In our study, the measurement of dietary intake data in a large, diverse, and nationally representative sample is a key asset to help establish the assumed link between dietary factors and noncommunicable diseases like hypertension in the general population, rather than certain study samples recruited from communities or medical centers. On top of that, the use of rigorous and standardized analytic approaches that conforms to the NHANES guidelines and full consideration of multiple factors with potential confounding effects assured that our analyses are reliable. Notwithstanding, there remained some limitations that need to be acknowledged: (1) due to the cross‐sectional nature of our study, we cannot infer the causality of association between vitamin A intake and adolescent hypertension; (2) since vitamin A intake was garnered from 24‐h dietary recall, our findings may be confounded by recall bias. Also, vitamin A intake is subject to seasonal factors because of the varying availability of vitamin A‐rich foods (usually plant‐based foods); however, lack of explicit data on the seasons when dietary interviews took place precluded us from assessing whether seasonal factor has a significant impact on our results; (3) despite great efforts to control for multiple confounders with known influence on adolescent hypertension, there might be some other unknown or unidentified confounding factors that have not been accounted for in our analyses. As such, further studies using longitudinal design or causal inference method such as Mendelian randomization are required to validate the relationship between vitamin A intake and adolescent hypertension.

## 5 Conclusion

Together, we revealed an inverse association between dietary intake of vitamin A and hypertension independently of multiple confounding factors in the US adolescents. A remarkable gender difference in the association was also found, in which boys can gain more benefits from vitamin A supplements in terms of preventing hypertension. Given the inherent limitations of cross‐sectional design, the causality of association needs to be verified by further research.

## Author Contributions

**Yukang Mao:** data curation (equal), investigation (equal), writing – original draft (equal), writing – review and editing (lead). **Lin Chen:** data curation (equal), investigation (equal), methodology (equal), writing – original draft (equal). **Yuli Wang:** methodology (equal), validation (supporting), visualization (equal). **Lida Wu:** software (lead), visualization (equal). **Guidong Xu:** formal analysis (supporting), validation (lead). **Xiangqing Kong:** conceptualization (equal), project administration (supporting), supervision (supporting). **Chao Chen:** conceptualization (equal), project administration (lead), resources (supporting). **Jiayi Weng:** conceptualization (equal), funding acquisition (equal), resources (lead), supervision (lead).

## Conflicts of Interest

The authors declare no conflicts of interest.

## Supporting information


**Table S1.** Baseline characteristics of study population grouped by quartiles of vitamin A intake.

## Data Availability

All supporting data relevant with the primary findings of this study are presented within the manuscript or deposited in the Supporting Information.
