# Supplementary material for: Association of Dietary Intake of Vitamin A With Adolescent Hypertension: A Cross‐Sectional Study Based on NHANES 1999–2018
Source: Food Sci Nutr. 2024 Dec 30;13(1):e4643. doi: 10.1002/fsn3.4643 (PMC11717040; doi:10.1002/fsn3.4643)
Supplement: Supplementary file 1 — Table S1. Baseline characteristics of study population grouped by quartiles of vitamin A intake. [file FSN3-13-e4643-s001.docx]

**Table S1. Baseline characteristics of study population grouped by quartiles of vitamin A intake**

| Vitamin A quantiles | Q1 | Q2 | Q3 | Q4 | *P* value |
| --- | --- | --- | --- | --- | --- |
| Age, % |  |  |  |  | <0.001*** |
| 10-14 years | 55.63 [52.93, 58.34] | 61.62 [59.50, 63.74] | 63.58 [61.29, 65.87] | 63.39 [61.45, 65.33] |  |
| 15-19 years | 44.37 [41.66, 47.07] | 38.38 [36.26, 40.50] | 36.42 [34.13, 38.71] | 36.61 [34.67, 38.55] |  |
| Gender, % |  |  |  |  | <0.001*** |
| Female | 60.20 [57.80, 62.60] | 53.37 [50.96, 55.77] | 48.54 [46.36, 50.71] | 39.56 [37.30, 41.81] |  |
| Male | 39.80 [37.40, 42.20] | 46.63 [44.23, 49.04] | 51.46 [49.29, 53.64] | 60.44 [58.19, 62.70] |  |
| Ethnicity, % |  |  |  |  | <0.001*** |
| Non-Hispanic White | 50.42 [46.67, 54.16] | 54.24 [50.95, 57.54] | 59.29 [56.34, 62.23] | 66.73 [63.88, 69.59] |  |
| Non-Hispanic Black | 21.35 [18.77, 23.93] | 15.75 [13.74, 17.76] | 13.18 [11.54, 14.81] | 9.01 [7.81, 10.22] |  |
| Mexican American | 13.12 [11.08, 15.16] | 15.53 [13.42, 17.64] | 12.89 [11.08, 14.70] | 10.90 [9.19, 12.62] |  |
| Other Hispanic | 6.87 [5.21, 8.53] | 6.99 [5.73, 8.26] | 6.09 [4.89, 7.28] | 6.18 [4.95, 7.41] |  |
| Others | 8.25 [6.53, 9.96] | 7.49 [6.10, 8.87] | 8.56 [7.12, 10.00] | 7.17 [5.99, 8.36] |  |
| Family income, % |  |  |  |  | <0.001*** |
| <$2000 | 22.94 [20.67, 25.22] | 17.77 [16.03, 19.51] | 15.80 [14.17, 17.42] | 13.29 [11.84, 14.73] |  |
| ≥$2000 | 77.06 [74.78, 79.33] | 82.23 [80.49, 83.97] | 84.20 [82.58, 85.83] | 86.71 [85.27, 88.16] |  |
| Energy, kcal | 1558.83 [1523.60, 1594.07] | 1879.42 [1848.16, 1910.69] | 2116.74 [2085.74, 2147.73] | 2584.64 [2541.05, 2628.22] | <0.001*** |
| SBP, mmHg | 108.06 [107.53, 108.58] | 106.83 [106.41, 107.25] | 106.37 [105.88, 106.87] | 106.14 [105.62, 106.67] | <0.001*** |
| DBP, mmHg | 60.19 [59.64, 60.74] | 59.36 [58.62, 60.10] | 58.75 [58.09, 59.42] | 59.00 [58.38, 59.62] | 0.01* |
| BMI, % |  |  |  |  | <0.001*** |
| Normal weight | 67.56 [65.03, 70.09] | 71.13 [68.96, 73.30] | 77.75 [75.95, 79.55] | 81.24 [79.40, 83.09] |  |
| Over weight | 14.86 [12.90, 16.81] | 11.80 [10.31, 13.28] | 8.93 [7.74, 10.12] | 6.57 [5.63, 7.52] |  |
| Obesity | 17.58 [15.60, 19.57] | 17.08 [15.30, 18.85] | 13.32 [11.92, 14.73] | 12.18 [10.63, 13.73] |  |
| eGFR, | 136.56 [135.37, 137.75] | 136.65 [135.53, 137.77] | 136.64 [135.52, 137.77] | 135.77 [134.63, 136.91] | 0.04* |
| FBG, mmol/L | 5.17 [5.13, 5.21] | 5.29 [5.21, 5.38] | 5.30 [5.14, 5.46] | 5.27 [5.18, 5.36] | <0.001*** |
| HbA1c, % | 5.19 [5.17, 5.21] | 5.22 [5.18, 5.25] | 5.21 [5.17, 5.25] | 5.19 [5.17, 5.21] | <0.001*** |
| DM, % | 0.68 [0.31, 1.05] | 1.10 [0.46, 1.74] | 0.97 [0.48, 1.46] | 0.85 [0.45, 1.24] | 0.61 |
| TG, mmol/L | 0.91 [0.88, 0.95] | 0.96 [0.90, 1.02] | 0.95 [0.90, 1.00] | 0.94 [0.89, 0.99] | 0.59 |
| TC, mmol/L | 4.13 [4.10, 4.17] | 4.09 [4.06, 4.13] | 4.12 [4.08, 4.15] | 4.06 [4.02, 4.10] | 0.03* |
| HDL-C, mmol/L | 1.32 [1.31, 1.34] | 1.35 [1.33, 1.36] | 1.35 [1.33, 1.37] | 1.32 [1.31, 1.34] | <0.001*** |
| LDL-C, mmol/L | 2.38 [2.32, 2.43] | 2.28 [2.24, 2.32] | 2.29 [2.22, 2.36] | 2.23 [2.19, 2.28] | <0.001*** |
| RBC, ×10^9^/L | 4.73 [4.71, 4.75] | 4.75 [4.72, 4.77] | 4.76 [4.74, 4.78] | 4.82 [4.80, 4.85] | <0.001*** |
| Hemoglobin, g/L | 13.78 [13.72, 13.85] | 13.85 [13.78, 13.91] | 13.94 [13.87, 14.00] | 14.12 [14.04, 14.21] | <0.001*** |

Continuous variables are presented as mean [95% CI], and category variables are presented as proportion [95% CI]. Comparisons of continuous and categorical variables among different quartiles of vitamin A intake were conducted using one-way ANOVA followed by a Bonferroni's *post hoc* test and chi-square test, respectively; **P* <0.05, ***P* <0.01, ****P* <0.001. Abbreviations: BMI, body mass index; DBP, diastolic blood pressure; DM, diabetes mellitus; eGFR, estimated glomerular filtration rate; FBG, fasting blood glucose; HbA1c, glycated hemoglobin; HDL-C, high-density lipoprotein cholesterol; LDL-C, low-density lipoprotein cholesterol; RBC, red blood cell; SBP, systolic blood pressure; TC, total cholesterol; TG, triglyceride.
